# Seasonal Epidemiology of Serum 25-Hydroxyvitamin D Concentrations among Healthy Adults Living in Rural and Urban Areas in Mongolia

**DOI:** 10.3390/nu8100592

**Published:** 2016-09-23

**Authors:** Sabri Bromage, Janet W. Rich-Edwards, Daria Tselmen, Ana Baylin, Lisa A. Houghton, Nachin Baasanjav, Davaasambuu Ganmaa

**Affiliations:** 1 Department of Nutrition, Harvard T.H. Chan School of Public Health, Boston, MA 02115, USA; 2 Connors Center for Women’s Health and Gender Biology, Brigham and Women’s Hospital, Harvard Medical School, Boston, MA 02115, USA; jwrichedwards@bics.bwh.harvard.edu; 3 National Institute of Medical Sciences, Ulaanbaatar 16081, Mongolia; tsemena312@gmail.com (D.T.); nachinbsj@yahoo.com (N.B.); 4 Departments of Epidemiology and Nutritional Sciences, University of Michigan School of Public Health, Ann Arbor, MI 48109, USA; abaylin@umich.edu; 5 Department of Human Nutrition, University of Otago, Dunedin 9054, New Zealand; lisa.houghton@otago.ac.nz; 6 Channing Division of Network Medicine, Department of Medicine, Brigham and Women’s Hospital and Harvard Medical School, Boston, MA 02115, USA; gdavaasa@hsph.harvard.edu

**Keywords:** 25(OH)D, 25-hydroxyvitamin D, calciferol, food fortification, hypovitaminosis D, Mongolia, nutritional epidemiology, sex differences, nutrient supplementation, vitamin D deficiency

## Abstract

Many factors put Mongolians at risk of vitamin D deficiency. Despite low levels observed in Mongolian children and pregnant women, there are few data published on the vitamin D status of non-pregnant adults. Between summer 2011 and winter 2013, paired summer and winter blood samples were collected from 320 healthy men and women (20–58 years) living in eight Mongolian provinces. Mean serum 25(OH)D concentrations were 22.5 ng/mL (95% CI: 14.5, 32.5) in summer and 7.7 ng/mL (95% CI: 4.6, 10.8) in winter, with a distribution (<10/10–20/20–30/≥30 ng/mL) of 3.1%/39.3%/39.6%/17.9% in summer and 80.1%/19.5%/0.3%/0.0% in winter. Residents of the capital, Ulaanbaatar, had lower levels in both seasons than any other region, whereas residents of the Gobi desert had the highest. In summer, indoor workers had significantly lower levels than outdoor workers (−2.3 ng/mL; 95% CI: −4.1, −5.7) while levels in males exceeded those in females (4.0 ng/mL; 95% CI: 2.3, 5.7). Effects of region, occupation, and sex were also significant in multivariable regression. In conclusion, Mongolian adults had extremely low serum 25(OH)D, particularly in winter, when 80.1% had concentrations below 10 ng/mL. These results indicate a need for effective vitamin D interventions for the Mongolian adult population, particularly among women and residents of Ulaanbaatar.

## 1. Introduction

Most of Mongolia lies north of the 42nd parallel, above which the average daily angle of incident solar radiation is too small to induce appreciable cutaneous synthesis of pre-vitamin D between November and March [1]. The fact that most of Mongolia is elevated above 1000 m means that the country’s surface temperature remains relatively depressed, especially during winter, because Mongolia is landlocked and is less shielded from extreme variations in climate that would otherwise be tempered by proximity to an ocean [2]. Cold weather results in significantly decreased ultraviolet-B (UV-B) exposure in humans because it discourages outdoor activity and encourages wearing more clothing [3]. As such, Mongolia’s geographic factors have historically combined to expose its population to a high risk of vitamin D deficiency. There also exists a low availability of vitamin D-rich foods in Mongolia, such as fish, and there are currently no vitamin-D fortified foods [4]. One local milk producer, Gum, produced vitamin D-fortified milk on a small scale in Ulaanbaatar, but has recently discontinued it. Vitamin D supplementation programs for children have had challenges achieving desired coverage or compliance [5].

A high prevalence of rickets was first reported among Ulaanbaatar children in 1992 [6] (42.4% of children under five years of age having at least one sign). Rickets had become drastically more prevalent in Mongolia following the country’s independence in 1990, and the halt of widespread pediatric supplementation programs. It has begun to decline very gradually over the following decade as supplementation programs have been restarted [7]. The most recent assessment of rickets and vitamin D status among Mongolian children is found in the nationally-representative Fourth National Nutrition Survey (FNNS) completed in 2010 by the Ministry of Health [5,8]. The FNNS found a considerable prevalence of classic rickets symptoms, including cranial deformation (18.3% of all children less than five years old deformed), pectus carinatum (pigeon chest) (9.5%), and genu varum (bow-leggedness) (15.7%). FNNS results from samples collected in September 2010 also revealed that 42.4% of children under five years of age had 25(OH)D serum concentrations below 10 ng/mL.

The vitamin D status of non-pregnant, reproductive age women was also investigated in the FNNS revealing that 52.2% of 867 women surveyed nationwide between July and September had serum 25(OH)D concentrations below 10 ng/mL [5]. Previous work from our group showed 79.3% of 420 women in Ulaanbaatar to have levels <10 ng/mL in spring [1]. Despite these concerning observations, epidemiologic studies of vitamin D status among Mongolian women have been solely descriptive in nature, and only one has accounted for seasonal variation; data from a study by Uush determined mean 25(OH)D levels in 62 Ulaanbaatar pregnant women during the summer (12.7 ng/mL), fall (11.7 ng/mL), winter (9.7 ng/mL), and spring (7.7 ng/mL) of 2000–2001 [9]. No studies have been conducted to determine the vitamin D status of Mongolian men, or to differentiate status between urban and rural areas or indoor and outdoor workers. Although Mongolia has a well-established infrastructure for public health practice and education, research funding is limited and assays for micronutrient analysis are not widely available to the public sector. Thus, data on micronutrient status and intake of Mongolian adults are limited or non-existent. As a consequence, the Ministry of Health lacks the necessary information to inform public policy regarding supplementation and food fortification programs to ensure micronutrient sufficiency throughout the country.

To respond to this dearth of information, the Nationwide Micronutrient Assessment of Mongolian Adults (a.k.a. “Nationwide Study”) was initiated in 2011 as a collaborative effort between the Harvard T.H. Chan School of Public Health, the Mongolian Institute of Medical Sciences, the Mongolian National University of Medical Sciences, and the University of Otago, New Zealand. Our specific objectives were to determine the seasonal status of vitamin D in adult subgroups defined by region, occupation, and sex, and to identify seasonal predictors of vitamin D status. We collected diet and lifestyle questionnaire data and paired summer and winter serum samples from 320 participants across eight regions in Mongolia between 2011 and 2013.

## 2. Materials and Methods

### 2.1. Study Population

The primary determinant of 25(OH)D in Mongolia was assumed to be exposure to UV-B radiation. The sampling frame was designed to maximize contrasts in UV-B exposure by sampling based on the season of assessment, geographic region of residence, indoor vs. outdoor occupation, and sex. Season was expected to be the most important of these variables, given the effects of season on the average daily hours of sunlight exposure, average intensity of UV-B exposure, and percentage of total body surface area exposed outdoors [10]. Region was also expected to have pronounced effects on UV-B exposure due to regional variations in latitude, altitude, air pollution, other atmospheric variables, and lifestyle factors [11]. Occupation was predicted to have a significant effect due to differences in the amount of average daily UV-B exposure incurred by “indoor” occupations (largely office workers), and “outdoor” occupations (largely nomadic herders). While sex was not expected to inherently affect 25(OH)D levels to a significant extent, it was hypothesized that pronounced differences in UV-B exposure might result from gender-based work patterns, as has been observed in other countries [11].

The regions selected for the study were the capital city of Ulaanbaatar, the southern province of Omnogobi, the north-central province of Bulgan, the northern province of Khuvsgul, the central province of Tuv, the northeastern province of Sukhbaatar, the western province of Khovd, and the eastern province of Dornod (Figure S1). Each region was sampled in both the summer and winter seasons between 2011 and 2013. Forty participants were recruited from each region, providing a total of 320 participants. Half of each of the regional groups of participants (*n* = 20 of 40) were composed of indoor workers (office workers, other white-collar professionals, and factory workers drawn from the urban areas of Ulaanbaatar and the provincial capitals of Dalanzadgad (Omnogobi), Bulgan (Bulgan), Murun (Khuvsgul), Zunmod (Tuv), Baruun Urt (Sukhbaatar), Khovd (Khovd), and Choibalsan (Dornod)). The remaining participants at each site were composed of outdoor workers (outdoor laborers drawn from Ulaanbaatar and herders drawn from the rural areas of Bayandalai (Omnogobi), Archon (Bulgan), Chagall (Khuvsgul), Altanbulag (Tuv), Khalzan (Sukhbaatar), Buyant (Khovd), and Bayantumen (Dornod)). Each group of indoor or outdoor workers included 10 males and 10 females. For each group of ten men or women, the margin of error for estimating the true mean summer or winter 25(OH)D level was estimated to be 5% [12].

The study regions were sampled based on their geographic disparateness. The urban and rural areas within each region were sampled by convenience, and eligible study participants within these areas were randomly sampled from a list of local residents. Eligible study participants were identified and located by local public health officials at each site, approached at their homes or work places, and asked if they would like to join the study. Participants were included in the study if they were 20 to 58 years old, free of acute or chronic health conditions, not pregnant, and able to participate in both summer and winter data collection. Ethical approval was obtained from the Mongolian Ministry of Health Ethical Review Board and the Harvard T.H. Chan School of Public Health Institutional Review Board (Protocol Title: “Nationwide study on Vitamin D and other micronutrients status among Mongolian adults”; Protocol Number: 21002; Submission Number: CR-21002-03). Eligible participants provided written informed consent.

### 2.2. Data Collection

Study visits were conducted between the months of June to August and January to March over three consecutive years from 2011 to 2013. Two 8 mL blood samples (winter and summer) were drawn from each participant into four vacutainers. The blood samples were then separated by centrifuge, and the serum was extracted and aliquoted. Serum aliquots were transported to Ulaanbaatar in a portable freezer where they were stored at −25 °C until analysis. Serum 25(OH)D analysis was conducted at the Bayangol Medical Center Clinical Laboratory using the DiaSorin LIAISON method [13], a direct competitive, chemiluminescence immunoassay using directly coated magnetic microparticles. To validate the LIAISON assay for use in this study, the investigators helped Bayangol laboratory to participate in the internationally-recognized Vitamin D External Quality Assessment Scheme (DEQAS) [14]. Validity was assessed according to DEQAS criteria throughout multiple validation runs spanning the duration of the study, by calculating the percent difference between our analyzed values of the 25(OH)D concentrations of the 40 DEQAS samples sent to our laboratory and their all-laboratory trimmed mean (ALTM) values assigned by DEQAS. Analyzed concentrations of 34 of the 40 DEQAS samples sent to our laboratory fell within 25% of the ALTM, satisfying the 80% standard required for DEQAS certification. A graphical comparison between the true and analyzed concentration of each sample is presented in Figure S2. For 20 of the DEQAS samples, the all-lab trimmed mean was in turn validated against values obtained by National Institute of Standards and Technology (NIST) reference measurement procedures, which yielded an *R*^2^ of 0.967 and a mean percent difference of −2.3%.

At the time of each blood collection, study participants were also administered a questionnaire by trained study staff members. As the same participants were sampled in both summer and winter, certain variables were assessed only in the summer as these variables were assumed to remain approximately constant throughout the six months between data collection periods. Variables assessed only once included each participant’s occupation, worksite, ethnicity, age, highest level of education attained, type of housing, and self-reported height and weight. Variables assessed in both summer and winter included the frequency of consumption of eggs, organ meats, and fish, use of supplements, sunscreen, a brimmed hat, and makeup, occurrence of sunburn, and source of milk and culinary flour (as locally-produced milk and flour are being considered as potential vehicles for vitamin D fortification in Mongolia). In addition, sun exposure defined as more or less than 30 min over five time periods of the day (9:00 to 11:00, 11:00 to 14:00, 14:00 to 16:00, 16:00 to 18:00, and after 18:00) in eight areas of the body (face, neck, torso, upper arms, lower arms, hands, upper legs, and lower legs) were assessed on separate week and weekend days

### 2.3. Data Variables

Body mass index (BMI) (kg/m^2^) was derived using each participant’s self-reported height and weight. Categorical variables of fish consumption, use of supplements, sunscreen, a brimmed hat, and makeup, occurrence of sunburn, and flour type were condensed into binary variables for use in regression models, and highest level education attained was also converted to a continuous variable of years of education attained based on the structure of the Mongolian education system. Total dietary intake of vitamin D (in international units (IU)/day) from eggs, organ meats, and fish was calculated using food composition data [15,16].

In summer and winter, the percent of body surface area (% BSA) typically exposed to the sun was calculated by summing the % BSA of each body part derived from a Lund-Browder chart [17] (a clinical tool for assessing burn severity) which was modified using more precise estimates of body part surface area from 3-D whole-body scans in the Taiwanese Bodybank [18]. A separate exposure duration score was calculated by summing up respondents’ periods of the day typically exposed, in which weekdays and weekends were weighted in a 5:2 ratio, “more than 30 min exposed” and “less than 30 min exposed” in a particular period of the day were weighted in a 2:1 ratio, and only exposure periods from 11:00 to 14:00 and 14:00 to 16:00 were considered as contributive to vitamin D status (we determined that incident radiation is not intense enough to induce vitamin D synthesis in Mongolia in the summertime during any other periods of the day using data from the Tropospheric Ultraviolet and Visible (TUV) Model [19]. Values of the score range from 0 (indicating no appreciable sun exposure) to 28 (indicating more than 30 min of exposure during both 11:00 to 14:00 and 14:00 to 16:00 on both weekdays and weekdays), where a 1 unit increase in score can be interpreted as the effect of increasing one’s exposure during either 11:00 to 14:00 or 14:00 to 16:00 from either “none” to “less than 30 min exposed” or from “less than 30 min” to “more than 30 min” on a single day of the week.

### 2.4. Statistical Analysis

Univariate analyses were conducted to describe the characteristics of participants, examine variable distributions, and detect missing values. The mean summer and winter serum 25(OH)D concentrations were calculated for subgroups of the study population according to the sampling scheme of season, region, occupation, and sex. The proportion of different subgroups’ subjects falling within different ranges of serum 25(OH)D concentrations (<10 ng/mL, 10 to <20 ng/mL, 20 to <30 ng/mL, and ≥30 ng/mL [20]) were also calculated. Data for two outlying participants in summer and one in winter were excluded from analyses (>50 ng/mL in summer or >25 ng/mL in winter). LIAISON measurements for one subject in summer and 12 subjects in winter fell short of the assay’s minimal detection limit of 4 ng/mL; in statistical analyses, these measurements were rounded to 3.9 ng/mL. Sensitivity analyses including excluded or rounded values did not materially affect results.

Within each season, differences in non-seasonal population characteristics between indoor and outdoor participants, and in seasonal variables between indoor and outdoor workers were compared using independent samples *t*-tests for continuous variables and chi-square tests for categorical variables (Fisher’s exact tests were used for categorical variables in which any cell counts were smaller than 5). A paired samples *t*-test was used to assess the difference in the mean 25(OH)D concentration between seasons. Within each season, differences in 25(OH)D levels between regions, and between occupation-sex groups, were assessed using ANOVA and Tukey-Kramer adjustment for multiple comparisons. Also within each season, independent samples t-tests were used to assess differences between indoor and outdoor workers across all regions, males and all females across all regions, indoor and outdoor workers within each region, and males and females within each of the 16 region-occupation groups.

In each season, measured serum 25(OH)D concentration was modeled as a linear function of each predictor in simple linear regression to identify significant risk factors for vitamin D deficiency. To improve the normality of residuals in the models, 25(OH)D concentrations were transformed by the natural logarithm. Parameter estimates and 95% confidence limits from the log-normal models were back-transformed to the original scale using the formula %ΔY = (e^β^-1) × 100, in which %ΔY represents the expected percent difference in 25(OH)D associated with a one-unit change in β [21]. The following three multiple regression models were run for each season in order to identify independent predictors of vitamin D status within each season: seasonal vitamin D serum concentration as a function of (1) age and sex; (2) age, sex, region, and occupation; and (3) age, sex, region, occupation, and any other statistically significant predictors of vitamin D status or apparent confounders of the association between age, sex, region, or occupation on vitamin D status. To improve interpretability of parameter estimates across seasons, the same (or seasonal-equivalent of) variables were used in both summer and winter models. Missing values in multiple regression were modeled using missing indicators in order to minimize the amount of statistical information lost.

Statistical analyses were performed using SAS version 9.4 (SAS Institute Inc., Cary, NC, USA). Alpha of 0.05 was used to determine significance of all statistical tests. Results are generally expressed as means ± standard deviations (SDs), or percentages.

## 3. Results

Non-seasonal demographic characteristics of the study population stratified by occupation are presented in Table 1. Overall, the study population was predominantly of Khalkh descent (82%), the ethnic majority of Mongolia. Participants were generally middle-aged (39.0 ± 9.7 years) ranging from 20 to 58 years old. On average, participants had completed 12.5 ± 3.8 years of education, with 12 years corresponding to the completion of high school in Mongolia. Outdoor occupation participants lived primarily in yurts (83%) and houses without central heating (14%), and indoor participants were more loosely distributed across different housing types. Indoor participants were primarily office workers (90%) and outdoor participants were mostly professional herders (86%) and urban or peri-urban outdoor laborers (13%). Table 2 presents participant characteristics that vary by season. Sunburn was more common in summer (80%) than in winter (50%), and use of a brimmed hat was more common in winter (70%) than summer (58%) among indoor participants. Vitamin D-containing supplement use did not vary between summer (19%) and winter (20%); only seven participants reported using vitamin D-containing supplements in any season. Most participants exclusively used Mongolian flour in their cooking (84% in summer and 72% in winter). The mean daily vitamin D intake from foods was less than 70 IU in any season.

**Figure 1:**
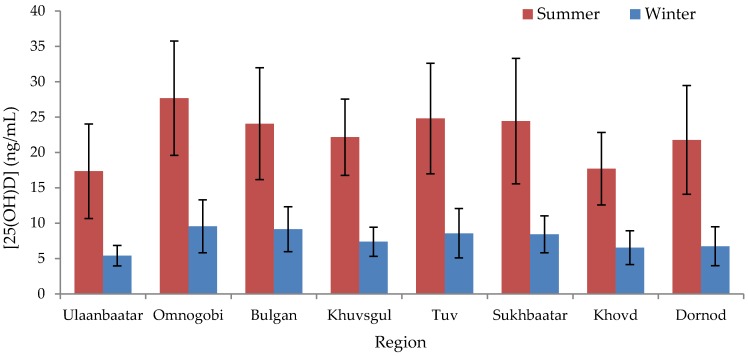
Mean (standard deviation, SD) serum 25(OH)D concentration by season and region. Bars indicate mean measured 25(OH)D concentrations (ng/mL) in summer (red bars) and winter (blue bars) ± SDs, summer *n* = 318, winter *n* = 307. *p* values for regional differences are provided in Table S2.

**Figure 2:**
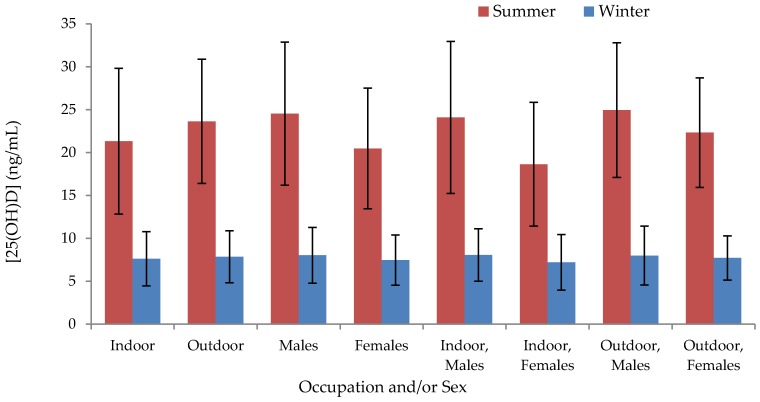
Mean (SD) serum 25(OH)D concentration by season, occupation, and sex. Bars indicate mean measured 25(OH)D concentrations (ng/mL) in summer (red bars) and winter (blue bars) ± SDs, summer *n* = 318, winter *n* = 307. Summer: Indoor < Outdoor, Males > Females, Indoor, Females < Indoor, Males = Outdoor, Males = Outdoor, Females, *p* < 0.05. Winter: Indoor = Outdoor, Males = Females, Indoor, Males = Indoor, Females = Outdoor, Males = Outdoor, Females, *p* < 0.05.

**Figure 3:**
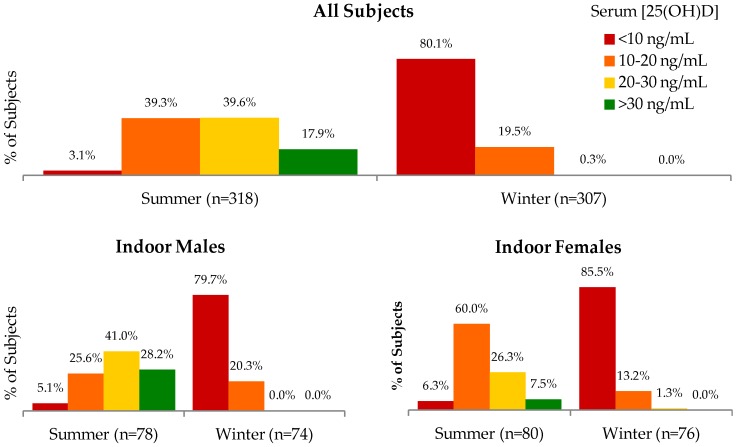

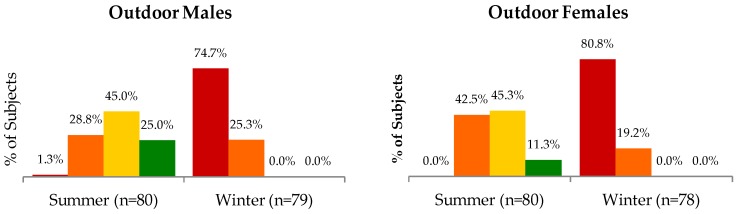
Prevalence of serum 25(OH)D concentration categories according to season, occupation, and sex subgroups (red: <10 ng/mL, orange: 10 to 20 ng/mL, gold: 20 to 30 ng/mL, green: >30 ng/mL).

The mean serum 25(OH)D concentration in Mongolian adults was 7.7 ± 3.1 ng/mL in winter and 22.5 ± 8.0 ng/mL in summer (difference of −14.9 ng/mL; 95% CI: −15.7, −14.1; *p* < 0.001) (Table S1). Only three participants’ winter serum 25(OH)D concentrations exceeded their summer concentrations. Vitamin D levels were unavailable for 12 participants in winter; the mean of the summer values for these 12 individuals was not significantly different from that of the rest of the group (*p* = 0.16). The correlation between summer and winter 25(OH)D values was 0.48. Residents of Ulaanbaatar exhibited a lower mean serum 25(OH)D concentration than those of any other region in summer (17.3 ± 6.7 ng/mL) and winter (5.4 ± 1.3 ng/mL), whereas those of Omnogobi exhibited the highest (27.7 ± 8.1 ng/mL in summer and 9.6 ± 3.7 ng/mL in winter) (Figure 1, Table S1), although only certain regional comparisons with Ulaanbaatar and Omnogobi were statistically significant (Table S2). In summer, indoor workers exhibited significantly lower serum concentrations than outdoor workers (−2.3 ng/mL; 95% CI: −4.1, −5.7; *p* = 0.010) and concentrations in males exceeded those in females (4.0 ng/mL; 95% CI: 2.3, 5.7; *p* < 0.001) (Figure 2, Table S1); differences between occupations or between males and females were not significant in winter. The proportion of subjects categorized according to different ranges of serum 25(OH)D concentrations are shown in Figure 3.

We next assessed differences in seasonal 25(OH)D levels between occupation groups within regions, and between males and females within regional occupation groups. Within regions, indoor workers had lower summer 25(OH)D concentrations than outdoor workers in six of eight regions, with statistically significant differences found in Sukhbaatar (−9.7 ng/mL; 95% CI: −14.5 to −4.9; *p* < 0.001) and Khovd (−5.2 ng/mL; 95% CI: −8.0, −2.3; *p* < 0.001) (Table S1); Sukhbaatar indoor workers also had significantly lower levels than herders in winter (−1.7 ng/mL; 95% CI: −3.4, −0.1; *p* = 0.042). With the exception of outdoor workers in Ulaanbaatar, the serum concentrations of males within every regional occupation group exceeded those of females in the summer (Table S1), though this difference was only statistically significant for Tuv indoor workers (8.3 ng/mL; 95% CI: 0.8, 15.7; *p* = 0.032); such a pattern was not observed in the winter.

Results of bivariate and multivariable regression models are summarized in Table 3 and Table 4, respectively, in which β is interpreted as the expected percent change in 25(OH)D levels in response to a one unit increase in a continuous parameter or a one level increase in a categorical parameter, holding other parameters constant. Consistent with the low intake and modest content of vitamin D naturally found in food, consumption of fish, liver, or eggs did not predict 25(OH)D levels in summer or winter, despite these being the best natural dietary sources of vitamin D in Mongolia. Supplement use was not associated with higher 25(OH)D levels in multivariable models, although models lacked statistical power, as supplement use was rare. Multivariable-adjusted results showed summer 25(OH)D levels were independently related to younger age, male sex, outdoor occupation, and residence outside Ulaanbaatar (except in Khovd). Although statistically significant, greater body surface exposure and less exposure duration had a clinically small impact on 25(OH)D levels (5.9% increase in 25(OH)D for a 1 SD increase in %BSA, and a −5.5% decrease with a 1 SD increase in duration of exposure). In winter, 25(OH)D levels were higher among younger individuals, men, and those residing outside of Ulaanbaatar, but other variables did not predict 25(OH)D levels.

## 4. Discussion

The present study indicates that Mongolian adults had extremely low vitamin D levels, particularly over the winter months, among office workers, and among women. We also observed significant regional variation in 25(OH)D levels, with the lowest concentrations in Ulaanbaatar and the highest in the Omnogobi region, in both winter and summer. This agrees with the latitude and climate of the Gobi desert and their resultant influence on the intensity and frequency of sun-exposure, respectively. Lower vitamin D status in Ulaanbaatar may be related to the fact that Ulaanbaatar suspended the second-highest annual average concentration of PM10 particulate matter pollution of all cities in the world in 2008 [22]. Air pollution has been associated with hypovitaminosis D in other parts of the world [23]. Ulaanbaatar residents may spend much more time indoors than those in less urbanized or rural areas. The fact that outdoor work was predictive of vitamin D status in both unadjusted and adjusted analyses is likely partly due to the fact that herders spend a large amount of time outdoors. However, this finding also remained after controlling for %BSA and exposure duration score, so it is difficult to attribute the beneficial association seen in herders to differences in sun-exposure behaviors alone.

From a nationally-representative sample of non-pregnant, 15–49 years old women surveyed from July to September, the Fourth National Nutrition Survey (FNNS) estimated the prevalence of vitamin D deficiency (serum 25(OH)D concentration <7.2 ng/mL) and low vitamin D reserve (7.2 to 9.6 ng/mL) to be 30.0% and 22.2%, respectively [5]. If our data are reanalyzed using the same categories as the FNNS, we estimate the summer/winter prevalence of vitamin D deficiency and low vitamin D reserve among women in our sample to be 0.6%/55.2% and 1.9%/27.3%, respectively. Our results are not immediately comparable with those of the FNNS for several reasons, including major differences in vitamin D assays, age distributions, sample frames, and seasons of measurement. Given that the FNNS’ deficiency and low reserve estimates lie between the corresponding summer and winter estimates of our study, it is possible that had the FNNS been conducted from June to August and January to March, estimates more comparable to those of our study would have been obtained. In both the FNNS and in reanalysis of our data using the FNNS definitions of deficiency and low reserve, no significant differences were found in the prevalence of either indicator between urban and rural women or across 10 years age categories. However, treatment of vitamin D status and age as continuous variables in our study has revealed significant effects of both urban/rural designation and age in both bivariate and multivariable analysis. The negative effect of age on vitamin D status is likely mostly attributable to changes in daily activity patterns and time spent outdoors as one grows older, but may also partly reflect reduced endogenous conversion of cholecalciferol to 25(OH)D [11]. The negative association with female sex has also been observed in other studies and may indicate a combination of biological and behavioral differences between men and women [11]. Other studies have observed a negative effect of BMI on vitamin D status, which may indicate greater sequestration of vitamin D in the adipose tissue of fatter individuals [10], however this was not observed in this population. Independent associations were also not found between vitamin D status and dietary intake of vitamin D-containing foods, or categorical sun exposure metrics such as sunburn and use of sunscreen, a brimmed hat, or makeup, though these variables have been implicated as predictors in other studies. The null effect of dietary intake on 25(OH)D levels reflects both the lack of vitamin D rich foods and their infrequent consumption. Total vitamin D intake from eggs, organ meats, and fish, at 20–40 IU daily, was far too low to impact circulating 25(OH)D levels; without fortification, it would likely be impractical to rely on dietary modification alone to improve population status in Mongolia. For reference, a scrambled egg, slice of beef liver, and half tin of oily fish (such as sprats) contain approximately 40, 33, and 200 IU of vitamin D, respectively.

One of the strengths of the study was the within-person seasonal assessment of vitamin D status together with the near-complete follow up. Ninety-six percent of participants sampled in summer were also sampled during the following winter. This is remarkable given the difficulty in relocating some of the herders in winter, as many of them had moved from their original locations, as well as the logistic difficulties posed by snow in the rural areas. By measuring vitamin D status of the same individuals in summer and winter, the estimated difference in summer and winter mean 25(OH)D levels within each subgroup of the population is not affected by between-subject variation. Another important strength is the use of a reference method for vitamin D assessment in a limited-resource setting. The participation of the laboratory in DEQAS ensures the analytical reliability of 25(OH)D assay and constitutes an important step forward in local capacity for epidemiologic assessment of vitamin D status in Mongolia. The study also benefits from the use of an anthropometrically-validated method for determining %BSA.

Although a larger sample size might have identified predictors of even more subtle differences in 25(OH)D levels, the current sample allowed the detection of differences in 25(OH)D levels as small as 1% (for one year of age), which represents a difference between 10 ng/mL and 10.1 ng/mL or 30 ng/mL and 30.3 ng/mL. In terms of study design, sampling in winter and summer provided data during periods of extreme sun exposure. However, future studies are warranted to further characterize vitamin D status in spring and fall. Sampling during periods of extreme sun exposure has also not necessarily provided the maximum range of serum 25(OH)D status expected in this population as the concentration of circulating 25(OH)D does not equilibrate until about eight weeks after sun-exposure [24]; thus, peak 25(OH)D levels might occur just prior to fall, and nadir just prior to spring. Additionally, while %BSA exposed was positively associated with vitamin D levels in summer, it was negatively associated in winter. The winter finding is likely the result of chance. UV monitoring may have provided more accurate measurements of sun-exposure duration, however our research also corroborates a need for more sensitive sun-exposure questionnaires, which may be less expensively administered in epidemiologic studies but which have as yet rarely succeeded in capturing more than 40% of variation in vitamin D status in other populations [25].

Vitamin D status in Mongolia may be improved in a number of ways. Sun exposure should be encouraged to reduce the likelihood of deficiency during summer, however it is unlikely that either increased sun exposure nor consumption of foods naturally rich in vitamin D will be sufficient to ensure adequacy for most of the population. This is particularly true for the winter months, during which fortification may be necessary. At the moment, the only example of universal fortification in Mongolia is that of salt with iodine. Given Mongolia’s highly centralized wheat flour production system (10 mills process approximately 90% of the country’s domestically-produced flour [26]), the high consumption of wheat flour products, and the observed predominance of Mongolian flour versus imported flour, vitamin D fortification of wheat may be a sustainable, long-term strategy for improving vitamin D status in the general population. Wheat flour may also be simultaneously fortified with other nutrients shown to be deficient in the population, such as folic acid (a pilot study among 40 women and 80 children in Mongolia demonstrated effectiveness of wheat flour fortification in raising plasma folic acid levels in both women and children [27]). Industrial fortification of wheat flour may be effectively combined with fortification of commercially-produced milk (consumption of which was also greater than that of imported milk) and increased supplementation of groups at particular risk, including women and children.

## 5. Conclusions

Vitamin D status was extremely low among Mongolian working-age adults, particularly in winter, during which 80.1% had concentrations below 10 ng/mL. These results indicate the need for both improved screening and intervention in this population, and research should continue to evaluate the efficacy of vitamin D supplementation, fortification, and dietary modification in Mongolia.

## Figures and Tables

**Table 1:** Non-seasonal characteristics of study population.

Characteristic	Occupation		*p*
	Indoor (*n* = 160)	Outdoor (*n* = 160)	
Female sex	80 (50)	80 (50)	1.00
Ethnicity			0.021
Khalkh	125 (88)	96 (75)	
Zakhchin	13 (9)	24 (19)	
Other	4 (3)	8 (6)	
Age, years	37.5 ± 9.9	40.3 ± 9.3	0.011
BMI, kg/m^2^	26.0 ± 4.2	25.1 ± 3.7	0.031
Education, years	14.8 ± 2.2	10.1 ± 3.6	<0.001
Housing			<0.001
Yurt	24 (17)	106 (83)	
Apartment	39 (28)	4 (3)	
House (central heating)	40 (29)	4 (3)	
House (no central heating)	37 (26)	14 (11)	
Worksite			<0.001
Outdoor labor	1 (1)	20 (13)	
Office	143 (90)	2 (1)	
Herder	0 (0)	138 (86)	
Factory	6 (4)	0 (0)	
Other	9 (6)	0 (0)	

Values are *n* (%) or means ± SDs. Percentages are calculated after excluding missing values. Extent of missingness: ethnicity (16%), age (5%), body mass index (BMI) (6%), education (16%), housing (16%), worksite (<1%). *p* values are drawn from tests of differences between occupations within the same seasons.

**Table 2:** Seasonal characteristics of study population.

Characteristic	Season and Occupation					
	Summer (*n* = 160)			Winter (*n* = 160)		
	Indoor (*n* = 80)	Outdoor (*n* = 80)	*p*	Indoor (*n* = 80)	Outdoor (*n* = 80)	*p*
Serum [25(OH)D], ng/mL	21.3 ± 8.5	23.6 ± 7.2	0.010	7.6 ± 3.2	7.9 ± 3.0	0.52
Body surface area exposed, %	32.9 ± 14.5	33.0 ± 14.4	0.94	17.4 ± 12.2	14.9 ± 8.7	0.045
Exposure duration score ^1^	11.1 ± 8.2	23.9 ± 6.8	<0.001	11.9 ± 7.2	17.8 ± 9.9	<0.001
Sun-exposure behaviors ^2^						
Used a brimmed hat	83 (58)	102 (81)	<0.001	101 (70)	119 (77)	0.16
Used sunscreen	27 (24)	15 (16)	0.14	15 (21)	13 (16)	0.46
Used makeup ^3^	54 (77)	37 (63)	0.07	57 (76)	48 (62)	0.05
Experienced sunburn	115 (81)	95 (78)	0.53	71 (54)	70 (47)	0.23
At least 100 IU/day of vitamin D from ^4^						
Fish	1 (1)	0 (0)	1.00	0 (0)	0 (0)	0.28
Eggs	0 (0)	1 (1)	0.08	2 (3)	0 (0)	1.00
Organ meats	0 (0)	0 (0)	1.00	0 (0)	0 (0)	1.00
Total IU/day from fish, eggs, organs ^4^	24.5 ± 30.6	15.4 ± 28.8	0.41	33.1 ± 37.2	17.5 ± 23.7	0.015
Supplement use ^2^			<0.001			0.36
Multivitamin	15 (13)	0 (0)		9 (6)	7 (6)	
Vitamin D	1 (1)	0 (0)		5 (4)	1 (1)	
Other or unspecified	13 (11)	17 (14)		13 (9)	17 (14)	
None	89 (75)	103 (86)		113 (81)	100 (80)	
Milk consumption, cups/day ^5^						
Manufactured milk, Mongolian	0.4 ± 0.4	0.8 ± 0.8	0.003	0.4 ± 0.6	0.4 ± 0.5	0.91
Manufactured milk, imported	0.1 ± 0.2	0.1 ± 0.2	0.27	0.0 ± 0.0	0.0 ± 0.1	0.48
Fresh cow milk	0.5 ± 0.4	1.4 ± 0.6	<0.001	0.3 ± 0.5	0.7 ± 0.6	<0.001
Source of cooking flour			0.39			0.65
Always Mongolian	46 (79)	53 (88)		58 (70)	51 (75)	
Mostly Mongolian	7 (12)	4 (7)		15 (18)	11 (16)	
Half Mongolian, half imported	4 (7)	2 (3)		8 (10)	5 (7)	
Mostly imported	1 (2)	0 (0)		0 (0)	1 (1)	
Always imported	0 (0)	1 (2)		2 (2)	0 (0)	

Values are *n* (%) or means ± SDs. Percentages are calculated after excluding missing values. *p* values are drawn from tests of differences between occupations within the same seasons. ^1^ Range of exposure duration score: 0 to 28; ^2^ Sun-exposure behaviors and supplement use are assessed as “Ever during past six months”; ^3^ Makeup use assessed in females only; ^4^ IU: international unit; ^5^ Cup volume: 240 mL.

**Table 3:** Unadjusted percent difference in 25(OH)D concentration by exposure to demographic and seasonal factors.

Parameter	Season			
	Summer (*n* = 318) ^1^		Winter (*n* = 307) ^1^	
	%ΔY (95% CI)	*p*	%ΔY (95% CI)	*p*
**Region**				
Ulaanbaatar	ref (16.2 ng/mL)	<0.001	ref (5.2 ng/mL)	<0.001
Omnogobi	63 (41, 89)	<0.001	69 (47, 95)	<0.001
Bulgan	41 (22, 63)	<0.001	65 (44, 90)	<0.001
Khuvsgul	33 (15, 54)	<0.001	36 (18, 56)	<0.001
Tuv	46 (26, 70)	<0.001	54 (34, 77)	<0.001
Sukhbaatar	39 (20, 62)	<0.001	54 (33, 77)	<0.001
Khovd	5 (−9, 21)	0.53	18 (2, 36)	0.03
Dornod	26 (9, 46)	<0.001	20 (4, 38)	0.01
**Occupation**				
Indoor	ref (17.1 ng/mL)	<0.001	ref (6.9 ng/mL)	<0.001
Outdoor	15 (6, 25)	<0.001	3 (−5, 12)	0.46
**Sex**				
Male	ref (27.5 ng/mL)	<0.001	ref (7.9 ng/mL)	<0.001
Female	−16 (−23, −9)	<0.001	−6 (−13, 2)	0.13
Age, years	−1 (−1, 0)	<0.001	−0 (−1, 0)	0.06
**Ethnicity**				
Khalkh	ref (21.6 ng/mL)	<.001	ref (7.5 ng/mL)	<0.001
Non-Khalkh	−0 (−11, 12)	0.98	−3 (−13, 8)	0.55
**BMI, kg/m^2^^1^**	−1 (−2, 0)	0.17	1 (0, 2)	0.15
**Education level**				
Secondary school or less	ref (22.4 ng/mL)	<0.001	ref (7.4 ng/mL)	<0.001
High school	4 (−7, 17)	0.46	6 (−6, 19)	0.36
University or professional certification	−9 (−19, 1)	0.06	−3 (−13, 8)	0.56
**Housing type**				
Apartment	ref (18.6 ng/mL)	<0.001	ref (7.3 ng/mL)	<0.001
Yurt	29 (14, 45)	<0.001	8 (−4, 22)	0.20
House with central heating	5 (−9, 21)	0.52	−9 (−22, 5)	0.21
House without central heating	11 (−4, 27)	0.17	−5 (−18, 10)	0.49
**Sun-exposure ^2^**				
% body surface area exposed, 1 SD ^3^	5.3 (1, 10)	0.01	−1 (−5, 3)	0.70
Exposure duration score, 1 SD ^3^	4.7 (0, 9)	0.05	2 (−2, 6)	0.40
Used a brimmed hat	10 (0, 21)	0.04	−10 (−18, −1)	0.04
Used sunscreen	−12 (−22, 0)	0.06	−3 (−17, 14)	0.75
Used makeup ^4^	−7 (−18, 5)	0.24	−6 (−17, 6)	0.30
Experienced sunburn	2 (−8, 14)	0.73	−4 (−12, 5)	0.36
**Diet and supplements**				
Any fish consumption ^2^	−5 (−17, 9)	0.49	−9 (−22, 8)	0.28
Portions/day of liver or organ meats	−11 (−47, 49)	0.65	−2 (−22, 25)	0.90
Number of eggs per day	−14 (−30, 6)	0.16	4 (−15, 28)	0.72
IU/day from fish, eggs, organs ^5^	0 (0, 0)	0.34	0 (0, 0)	0.93
Used multivitamins or vitamin D ^2^	19 (−1, 43)	0.06	1 (−13, 18)	0.89

%ΔY for continuous parameters is interpreted as the % difference in seasonal 25(OH)D concentration associated with a one-unit increase in the parameter. %ΔY for levels of categorical variables are interpreted as the % change in seasonal 25(OH)D concentration associated with each level of the parameter. *p*-values are those associated with the difference in log(25(OH)D) for a one-unit change in a continuous parameter, a level of a categorical parameter relative to the reference category (ref), or the reference category itself. Reference category values are obtained from the model intercepts. ^1^ In summer, two vitamin D outliers were excluded (*n* = 318), and in winter, one vitamin D outlier was excluded and 12 vitamin D measurements were missing (*n* = 307); ^2^ Sun-exposure behaviors, supplement use, and fish consumption are assessed as “Ever during past six months”. %ΔY for these parameters is expressed in comparison to the reference group “never”; ^3^ %ΔY for % body surface area exposed and exposure duration score are expressed in terms of a 1 SD change in the parameter; ^4^ Makeup use assessed in females only; ^5^ IU: international unit.

**Table 4:** Multivariable adjusted percent differences in 25(OH)D concentration by exposure to demographic and seasonal factors.

Parameter	Season			
	Summer (*n* = 318) ^1^		Winter (*n* = 307) ^1^	
	%ΔY (95%CI) or Intercept	*p*	%ΔY (95%CI) or Intercept	*p*
**Model 1**				
Intercept	36 ng/mL	<0.001	9 ng/mL	<0.001
Age, years	−1 (−1, 0)	<0.001	−0 (−1, 0)	0.07
Female sex	−15 (−22, −9)	<0.001	-6 (-13, 2)	0.16
**Model 2**				
Intercept	23 ng/mL	<0.001	6 ng/mL	<0.001
Age, years	−1 (−1, −1)	<0.001	−1 (−1, 0)	<0.01
Female sex	−15 (−21, −10)	<0.001	−5 (−11, 2)	0.16
Region				
Ulaanbaatar	ref		ref	
Omnogobi	64 (44, 88)	<0.001	69 (47, 94)	<0.001
Bulgan	45 (27, 67)	<0.001	69 (47, 94)	<0.001
Khuvsgul	36 (18, 55)	<0.001	37 (19, 57)	<0.001
Tuv	49 (30, 70)	<.001	55 (35, 78)	<0.001
Sukhbaatar	45 (26, 66)	<0.001	56 (35, 79)	<0.001
Khovd	6 (−7, 22)	0.37	19 (3, 37)	0.02
Dornod	31 (15, 50)	<0.001	22 (6, 40)	<0.01
Outdoor occupation (vs. indoor)	17 (10, 26)	<0.001	4 (−3, 12)	0.24
**Model 3**				
Intercept	19 ng/mL	<0.001	5 ng/mL	<0.001
Age, years	−1 (−1, 0)	<0.001	0 (−1, 0)	<0.01
Female sex	−17 (−22, −11)	<0.001	−7 (−13, 0)	0.06
Region				
Ulaanbaatar	ref		ref	
Omnogobi	68 (45, 95)	<0.001	79 (53, 111)	<0.001
Bulgan	49 (27, 75)	<0.001	76 (53, 107)	<0.001
Khuvsgul	34 (17, 54)	<0.001	42 (25, 68)	<0.001
Tuv	54 (33, 78)	<.001	66 (43, 98)	<0.001
Sukhbaatar	39 (20, 62)	<.001	60 (40, 92)	<0.001
Khovd	9 (−7, 29)	0.29	31 (10, 59)	<0.01
Dornod	35 (14, 60)	<0.001	33 (13, 64)	<0.01
Outdoor occupation (vs. indoor)	23 (11, 35)	<0.001	7 (−2, 19)	0.11
% body surface area exposed, 1 SD ^2^	6 (2, 10)	<0.01	−0 (−4, 4)	0.95
Exposure duration score, per 1 SD ^2^	−6 (−11, 0)	0.04	1 (−3, 5)	0.71
Housing type				
Apartment	ref		ref	
Yurt	7 (−6, 21)	0.32	−9 (−21, 3)	0.12
House with central heating	4 (−9, 18)	0.61	−10 (−22, 3)	0.11
House without central heating	13 (−1, 29)	0.06	1 (−13, 14)	0.99

%ΔY for continuous parameters is interpreted as the % difference in seasonal 25(OH)D concentration associated with a one-unit increase in the parameter. %ΔY for levels of categorical variables are interpreted as the % change in seasonal 25(OH)D concentration associated with each level of the parameter. *p*-values are those associated with the difference in log(25(OH)D) for a one-unit change in a continuous parameter, a level of a categorical parameter relative to the reference category (“ref”), or the reference category itself. ^1^ In summer, two vitamin D outliers were excluded (*n* = 318), and in winter, one vitamin D outlier was excluded and 12 vitamin D measurements were missing (*n* = 307); ^2^ %ΔY for % body surface area exposed and exposure duration score are expressed in terms of a 1 SD change in the parameter.

## Supplementary Materials

The following are available online at http://www.mdpi.com/2072-6643/8/9/592/s1, Figure S1: Map of Mongolia with study regions highlighted; Figure S2: DEQAS validation of DiaSorin LIAISON Assay; Table S1: Mean (SD) serum 25(OH)D concentration by season, region, occupation, and sex; Table S2: Statistical significance of regional differences in mean serum 25(OH)D concentrations.
