# Supplementary material for: Seasonal Epidemiology of Serum 25-Hydroxyvitamin D Concentrations among Healthy Adults Living in Rural and Urban Areas in Mongolia
Source: Nutrients. 2016 Sep 23;8(10):592. doi: 10.3390/nu8100592 (PMC5083980; doi:10.3390/nu8100592)
Supplement: Supplementary file 1 [file nutrients-08-00592-s001.docx]

Supplementary Materials: Seasonal Epidemiology of Serum 25-Hydroxyvitamin D Concentrations among Healthy Adults Living in Rural and Urban Areas in Mongolia

Sabri Bromage, Janet W. Rich-Edwards, Daria Tselmen, Ana Baylin, Lisa A. Houghton, Nachin Baasanjav and Davaasambuu Ganmaa

**Table S1.** Mean (±SD) serum 25(OH)D concentration by season, region, occupation, and sex.

| **Season ^1^** | **Region ^2^** | **Occupation ^3^** | **Sex ^4^** | **Season ^1^** | **Region ^2^** | **Occupation ^3^** | **Sex ^4^** |
| --- | --- | --- | --- | --- | --- | --- | --- |
| Summer  22.5 ± 8.0 | Ulaanbaatar  17.3 ± 6.7 | Indoor  16.4 ± 8.3 | Male  19.7 ± 9.9 | Winter  7.7 ± 3.1 | Ulaanbaatar  5.4 ± 1.5 | Indoor  5.4 ± 1.3 | Male  5.7 ± 1.4 |
|  |  |  | Female  13.1 ± 5.0 |  |  |  | Female  5.1 ± 1.0 |
|  |  | Outdoor  18.3 ± 4.5 | Male  17.5 ± 3.3 |  |  | Outdoor  5.4 ± 1.7 | Male  5.4 ± 1.8 |
|  |  |  | Female  19.1 ± 5.5 |  |  |  | Female  5.5 ± 1.6 |
|  | Omnogobi  27.7 ± 8.1 | Indoor  29.7 ± 7.6 | Male  32.5 ± 6.2 |  | Omnogobi  9.6 ± 3.7 | Indoor  10.3 ± 3.5 | Male *  12.2 ± 3.2 |
|  |  |  | Female  26.9 ± 8.1 |  |  |  | Female  8.4 ± 2.8 |
|  |  | Outdoor  25.6 ± 8.3 | Male  29.1 ± 8.9 |  |  | Outdoor  8.8 ± 3.9 | Male *  11.0 ± 4.3 |
|  |  |  | Female  22.1±6.2 |  |  |  | Female  6.6± 1.8 |
|  | Bulgan  24.1 ± 7.9 | Indoor  23.8 ± 7.7 | Male  25.5 ± 6.1 |  | Bulgan  9.1 ± 3.2 | Indoor  8.7 ± 3.0 | Male  8.7 ± 2.8 |
|  |  |  | Female  22.3 ± 8.9 |  |  |  | Female  8.8 ± 3.3 |
|  |  | Outdoor  24.3 ± 8.3 | Male  26.4 ± 7.8 |  |  | Outdoor  9.5 ± 3.4 | Male  10.8 ± 3.7 |
|  |  |  | Female  22.2 ± 8.6 |  |  |  | Female  8.2 ± 2.5 |
|  | Khuvsgul  22.2 ± 5.4 | Indoor  23.1 ± 6.5 | Male *  27.0 ± 6.5 |  | Khuvsgul  7.4 ± 2.1 | Indoor | Male  7.7 ± 2.0 |
|  |  |  |  |  |  | 7.5 ± 1.6 |  |
|  |  |  | Female  19.7 ± 4.4 |  |  |  | Female  7.3 ± 1.1 |
|  |  | Outdoor  21.3 ± 4.0 | Male  21.3 ± 4.0 |  |  | Outdoor  7.3 ± 2.4 | Male  7.2 ± 2.5 |
|  |  |  | Female  21.2 ± 4.2 |  |  |  | Female  7.3 ± 2.5 |
|  | Tuv  24.8 ± 7.8 | Indoor  23.6 ± 8.8 | Male *  27.7 ± 9.6 |  | Tuv  8.6 ± 3.5 | Indoor  8.6 ± 1.7 | Male  7.9 ± 2.8 |
|  |  |  | Female  19.4 ± 5.8 |  |  |  | Female  9.2 ± 6.0 |
|  |  | Outdoor  26.0 ± 6.7 | Male  28.0 ± 6.0 |  |  | Outdoor  8.6 ± 1.7 | Male  8.0 ± 1.3 |
|  |  |  | Female  24.1 ± 7.1 |  |  |  | Female  9.2 ± 2.0 |
|  | Sukhbaatar  24.4 ± 8.9 | Indoor *  19.6 ± 6.1 | Male  21.5 ± 6.5 |  | Sukhbaatar  8.4 ± 2.6 | Indoor *  7.5 ± 2.3 | Male  7.6 ± 2.8 |
|  |  |  | Female  17.6 ± 5.4 |  |  |  | Female  7.6 ± 2.8 |
|  |  | Outdoor  29.3 ± 8.6 | Male  31.0 ± 9.9 |  |  | Outdoor  9.2 ± 2.7 | Male  8.8 ± 2.9 |
|  |  |  | Female  27.7 ± 7.3 |  |  |  | Female  9.7 ± 2.5 |
|  | Khovd  17.7 ± 5.1 | Indoor *  15.1 ± 4.3 | Male  16.2 ± 4.7 |  | Khovd  6.5 ± 2.4 | Indoor  6.4 ± 2.1 | Male  7.1 ± 2.6 |
|  |  |  | Female  14.1 ± 3.7 |  |  |  | Female  5.8 ± 1.3 |
|  |  | Outdoor  20.3 ± 4.7 | Male  21.5 ± 5.9 |  |  | Outdoor  6.6 ± 2.7 | Male  6.7 ± 3.4 |
|  |  |  | Female  19.1 ± 2.8 |  |  |  | Female  6.6 ± 2.0 |
|  | Dornod  21.8 ± 7.7 | Indoor  19.5 ± 8.9 | Male  23.1 ± 10.4 |  | Dornod  6.7 ± 2.8 | Indoor  6.1 ± 2.5 | Male  7.2 ± 3.5 |
|  |  |  | Female  16.0 ± 5.5 |  |  |  | Female  5.4 ± 1.3 |
|  |  | Outdoor  24.0 ± 5.7 | Male  24.9 ± 6.3 |  |  | Outdoor  7.3 ± 2.9 | Male **  6.0 ± 2.3 |
|  |  |  | Female  23.1 ± 5.1 |  |  |  | Female  8.6 ± 2.9 |

Values indicate the mean measured 25(OH)D concentration (ng/mL) in each subgroup ± SDs, summer *n* = 318, winter *n* = 307. ^1^ Summer > Winter, *p* < 0.05; ^2^ *p* values for regional differences are provided in Table S2; ^3,^* Within season and region, Indoor < Outdoor, *p* < 0.05; ^4,^* Within season, region, and occupation, Male > Female, *p* < 0.05. ** Within season, region, and occupation, Male < Female, *p* < 0.05.

**Table S2.** Statistical significance of regional differences in mean serum 25(OH)D concentration.

|  | **Ulaanbaatar** | **Omnogobi** | **Bulgan** | **Khuvsgul** | **Tuv** | **Sukhbaatar** | **Khovd** | **Dornod** |
| --- | --- | --- | --- | --- | --- | --- | --- | --- |
| Ulaanbaatar |  | <0.001 | 0.002 | 0.07 | <0.001 | <0.001 | 1.00 | 0.13 |
| Omnogobi | <0.001 |  | 0.36 | 0.021 | 0.65 | 0.50 | <0.001 | 0.009 |
| Bulgan | <0.001 | 1.00 |  | 0.94 | 1.00 | 1.00 | 0.003 | 0.86 |
| Khuvsgul | 0.044 | 0.017 | 0.11 |  | 0.75 | 0.86 | 0.12 | 1.00 |
| Tuv | <0.001 | 0.78 | 0.99 | 0.59 |  | 1.00 | <0.001 | 0.58 |
| Sukhbaatar | <0.001 | 0.65 | 0.96 | 0.73 | 1.00 |  | 0.001 | 0.73 |
| Khovd | 0.67 | <0.001 | 0.002 | 0.90 | 0.042 | 0.08 |  | 0.21 |
| Dornod | 0.43 | <0.001 | 0.005 | 0.98 | 0.09 | 0.16 | 1.00 |  |

Values indicate *p* values for within-season pairwise comparisons of mean measured 25(OH)D concentration between regions, applying Tukey-Kramer adjustment for multiple comparisons. Upper diagonal: summer; lower diagonal: winter.

**Figure S1.** Map of Mongolia with study regions highlighted. Values below the name of each province indicate the mean measured 25(OH)D concentrations (ng/mL) in summer/winter, summer *n* = 318, winter *n* = 307.

**Figure S2.** DEQAS validation of DiaSorin LIAISON assay (comparison of 40 analyzed values measured using DiaSorin LIAISON with DEQAS all-laboratory trimmed means for the same 40 samples).
